# Thyroid Incidentalomas on ^18^F-FDG PET/CT: Clinical Significance and Controversies

**DOI:** 10.4274/mirt.94695

**Published:** 2017-10-02

**Authors:** William Makis, Anthony Ciarallo

**Affiliations:** 1 Cross Cancer Institute, Department of Diagnostic Imaging, Edmonton, Canada; 2 MUHC Glen Site, Department of Nuclear Medicine, Montreal, Canada

**Keywords:** Thyroid incidentaloma, thyroid carcinoma, ^18^F-fluorodeoxyglucose, ^18^F-FDG, Positron emission tomography, PET, PET/CT

## Abstract

**Objective:**

The purpose of the current study is to examine the incidence and clinical significance of unexpected focal uptake of ^18^F-fluorodeoxyglucose (^18^F-FDG) on positron emission tomography/computed tomography (PET/CT) in the thyroid gland of oncology patients, the maximum standardized uptake value (SUV_max_) of benign and malignant thyroid incidentalomas in these patients, and review the literature.

**Methods:**

Seven thousand two hundred fifty-two ^18^F-FDG PET/CT studies performed over four years, were retrospectively reviewed. Studies with incidental focal ^18^F-FDG uptake in the thyroid gland were further analyzed.

**Results:**

Incidental focal thyroid ^18^F-FDG uptake was identified in 157 of 7252 patients (2.2%). Sufficient follow-up data (≥12 months) were available in 128 patients, of whom 57 (45%) had a biopsy performed and 71 had clinical follow-up. Malignancy was diagnosed in 14 of 128 patients (10.9%). There was a statistically significant difference between the median SUV_max_ of benign thyroid incidentalomas (SUV_max_ 4.8) vs malignant (SUV_max_ 6.3), but the wide range of overlap between the two groups yielded no clinically useful SUV_max_ threshold value to determine malignancy.

**Conclusion:**

^18^F-FDG positive focal thyroid incidentalomas occurred in 2.2% of oncologic PET/CT scans, and were malignant in 10.9% of 128 patients. This is the lowest reported malignancy rate in a North American study to date, and significantly lower than the average malignancy rate (35%) reported in the literature. Invasive biopsy of all ^18^F-FDG positive thyroid incidentalomas, as recommended by some studies, is unwarranted and further research to determine optimal management is needed. There was no clinically useful SUV_max_ cut-off value to determine malignancy and PET/CT may not be a useful imaging modality to follow these patients conservatively.

## Abstract (tr)

**Amaç:**

Bu çalışmanın amacı onkoloji hastalarının ^18^F-florodeoksiglukoz (^18^F-FDG) pozitron emisyon tomografisi/bilgisayarlı tomografisinde (PET/BT) tesadüfen saptanan tiroid bezi fokal tutulumunun insidansını ve klinik önemini ve bu hastalarda benign ve malign tiroid insidentalomalarının maksimum standardize tutulum değerini (SUV_maks_) değerlendirmek ve literatürü gözden geçirmektir.

**Yöntem:**

Dört yıl boyunca yapılmış 7252 ^18^F-FDG PET/BT incelemesi retrospektif olarak değerlendirildi. Tiroid bezinde insidental fokal ^18^F-FDG tutulumu olanlar incelemeye dahil edildi.

**Bulgular:**

7252 hastanın 157’sinde insidental fokal tiroid ^18^F-FDG tutulumu saptandı (%2,2). Yüz yirmi sekiz hastada yeterli takip verisi mevcuttu (≥12 ay), bunların 57’sine (%45) biyopsi uygulanmış ve 71’i klinik olarak takip edilmişti. Yüz yirmi sekiz hastanın 14’ünde malignite saptanmıştı (%10,9). Benign tiroid insidentalomalarının ortanca maksimum standardize tutulum değeri (SUV_maks_ 4,8) ile malign tiroid insidentalomalarının değerleri (SUV_maks_ 6,3) arasında istatistiki olarak anlamlı fark saptandı, ancak her iki grup arasındaki geniş aralıklı örtüşme olduğundan malignite saptamada klinik olarak yararlı olabilecek bir SUV_maks_ eşik değeri saptanmadı.

**Sonuç:**

^18^F-FDG pozitif fokal tiroid insidentaloması onkolojik PET/BT taramalarının %2,2’sinde görüldü ve 128 hastanın %10,9’unda malignite mevcuttu. Bu oran günümüze kadar Kuzey Amerika çalışmalarında bildirilen en düşük düzeydir ve literatürde bildirilen ortalama malignite oranından (%35) ciddi şekilde azdır. Bazı çalışmalarda önerildiği gibi tüm ^18^F-FDG pozitif tiroid insidentalomalarının invazif biyopsisi sağlam bir temele dayanmamaktadır, optimal yaklaşımı belirlemek için ileri çalışmalara ihtiyaç vardır. Malignite saptamak için klinikte kullanılabilecek bir SUV_maks_ eşik değeri bulunmamıştır, bu hastaları konservatif olarak takip etmek için PET/BT yararlı bir modalite olmayabilir.

## INTRODUCTION

One of the main challenges that face positron emission tomography/computed tomography (PET/CT) readers is the interpretation of foci of abnormal ^18^F-FDG uptake in unexpected anatomic locations (1,2,3,4,5,6,7,8,9,10). The thyroid gland is the best studied anatomic location of incidental ^18^F-FDG uptake, with well over 30 studies examining the clinical significance of thyroid incidentaloma (11-22), including 3 systematic reviews (11-13). However, thyroid incidentalomas still remain a source of controversy in the literature.

The malignancy rates in thyroid incidentalomas range in the literature from 10% up to 64%. Three systematic reviews have reported a pooled malignancy rate of 33-35%. One major point of contention with most of these studies is that only a small subset of patients is biopsied (usually patients with a high clinical suspicion of malignancy), and most thyroid incidentaloma patients are not investigated or followed further. The largest systematic review of thyroid incidentaloma studies (27 studies) revealed a biopsy rate of only 35%. Many papers in the literature recommend that all thyroid incidentaloma patients be invasively biopsied, however this recommendation is based on a malignancy rate derived from a subset of non-randomly selected thyroid incidentaloma patients.

There is also controversy over the utilization of SUV_max_ to differentiate benign from malignant thyroid incidentalomas. Many studies have made attempts to determine an optimal cut-off SUV_max_ value for differentiating benign from malignant lesions. Only half of these studies have managed to detect a statistically significant difference. Three meta-analyses reflect these conflicting results.

The purpose of this retrospective review was to determine the incidence of unexpected focal uptake of ^18^F-FDG in the thyroid gland of oncology patients (with no prior history of thyroid cancer) and what proportion of these cases were malignant. We also evaluated the feasibility of using SUV_max_ to identify malignant causes of incidental focal thyroid ^18^F-FDG uptake and investigated whether a clinically useful cut-off value of SUV_max_ could be determined.

## MATERIALS AND METHODS

A retrospective review of 7252 oncologic ^18^F-FDG PET/CT studies performed over the course of 48 months (January 1, 2006-December 31, 2009) was done. PET/CT studies with incidental focal ^18^F-FDG thyroid gland uptake, regardless of corresponding CT findings, formed the basis for this review.

One hundred fifty-seven (n=157) patients out of 7252 (2.2%) had unexpected focal ^18^F-FDG thyroid uptake and comprised the study group. We excluded patients who had a history of a previous thyroid malignancy or predisposing condition (e.g. Cowden syndrome) (n=6), and patients who had insufficient follow-up data (less than 12 months) (n=23). The remaining 128 patients comprised the study group that was evaluated further to determine the clinical significance of unexpected focal ^18^F-FDG thyroid gland uptake. The primary malignant diagnoses of these 128 patients are listed in Table 1.

### PET/CT Examination and Interpretation

The PET scanner used in this study was the Discovery ST with a 16-slice CT (GE Healthcare, WI, USA). ^18^F-FDG PET/CT exams were performed according to routine institutional protocol. After a minimum of 4 to 6 hours of fasting, patients waited in a quiet and dark room just prior to their exam. Serum glucose levels were tested and 0.22 mCi (8.14 MBq)/kg dose of ^18^F-FDG was injected (to a maximum dose of 20 mCi, or 740 MBq). Approximately 60 minutes after injection of ^18^F-FDG, PET portion of the study was acquired in 2D from the base of the skull to the proximal thighs. Data were acquired for 4-5 min per bed position (depending on patient body weight). CT portion of the study was acquired (140 kVp, 90-110 mA) with a rotation time of 0.8 s, pitch 1.75:1 and detector row configuration of 16x0.625 mm. The patient was breathing normally during both PET and CT acquisitions.

The PET data were reconstructed iteratively with ordered-subsets expectation maximization algorithm (21 subsets, 2 iterations). PET emission data were corrected for photon attenuation effects using CT images. PET/CT exams were interpreted by two nuclear medicine board certified physicians independently on a dedicated Xeleris 2.0 workstation (GE Healthcare, Waukesha, WI, USA). Any unexpected focal ^18^F-FDG thyroid gland uptake was noted and maximum standardized uptake value (SUV_max_ corrected for body weight) was measured using a spherical region of interest (ROI) at the site of most intense ^18^F-FDG accumulation.

### Diagnosis

Histopathologic evaluation or clinical follow-up (with or without serial PET/CT examinations) over a time period of at least 12 months determined the final diagnosis of either benign thyroid incidentaloma or malignant thyroid incidentaloma. Histological sampling was available in 57 of 128 patients and the other 71 patients were assessed clinically over a minimum period of 12 months or more, with a mean clinical follow-up time of 28 months (range: 12-70 months). Global clinical assessment comprised a physical examination and evaluation of all available biochemical and diagnostic imaging studies.

### Statistical Analysis

The Wilcoxon-Mann-Whitney test was used to compare the ^18^F-FDG PET/CT SUV between benign and malignant thyroid lesions. Numeric data were expressed as median ± interquartile range (IQR). P values of less than 0.05 were considered to indicate a statistically significant difference.

**Ethical statement:** The study was approved by an institutional review board or equivalent and has been performed in accordance with the ethical standards laid down in the 1964 Declaration of Helsinki and its later amendments. All subjects in the study gave written informed consent or the institutional review board waived the need to obtain informed consent.

## RESULTS

Out of 128 patients included in the study, there were 31 men and 97 women. One hundred fourteen (89.1%) were diagnosed with a benign thyroid process and 14 (10.9%) were diagnosed with a thyroid malignancy. The mean age of patients with benign thyroid lesions was 62.8 years, compared to 57.1 years for patients with malignant thyroid lesions. A total of 154 individual ^18^F-FDG positive thyroid lesions were identified in these 128 patients. The locations of the thyroid lesions are given in Table 2.

Histological evaluation was available in 57 of 128 patients. Fourteen of 57 were malignant (11 papillary, 1 follicular, 1 lymphoma and 1 metastasis) and 43 of 57 were benign (23 Hurthle cell metaplasia, 11 nodular goiter, 5 benign epithelium, 2 thyroiditis, 2 follicular adenoma) (Table 3). The mean SUV_max_ of each lesion type is given in Table 3.

The remaining 71 patients were followed clinically. In addition to global clinical assessment, PET/CT follow-up studies were available in 29 of 71 patients, and the results of follow-up PET/CT SUV_max_ of incidental focal thyroid uptake are summarized in Table 4.

There was a statistically significant difference between the median SUV_max_ values in benign thyroid incidentalomas (median SUV_max_ 4.8) versus malignant lesions (median SUV_max_ 6.3), p value=0.03, Wilcoxon Mann Whitney-U test (Table 5). However, there was a significant overlap between the ranges of benign and malignant thyroid incidentalomas with benign SUV_max_ values ranging from 2.1-30.5, and malignant SUV_max_ values ranging from 3.4 to 28.1.

A few potential SUV_max_ cut-offs were examined and a kappa statistic was calculated for each value to see which would maximize sensitivity and specificity. The SUV_max_ cut-off with the highest kappa coefficient is provided (Table 6). These calculations were performed to determine if there was a satisfactory SUV_max_ cut-off to differentiate benign thyroid lesions from malignant ones.

**Figure 1:**
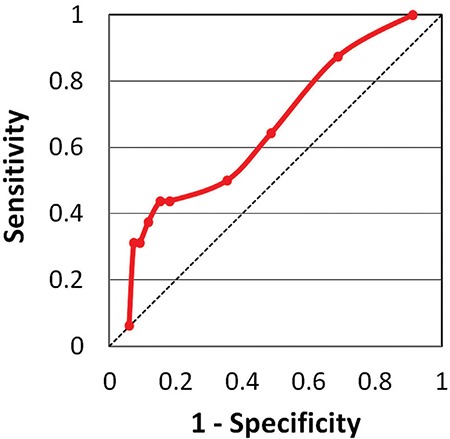
Receiver-operating-characteristic curve with sensitivity (y-axis) versus 1-Specificity (x-axis) shows no satisfactory value for SUV_max_ to differentiate benign from malignant thyroid incidentalomas

**Figure 2:**
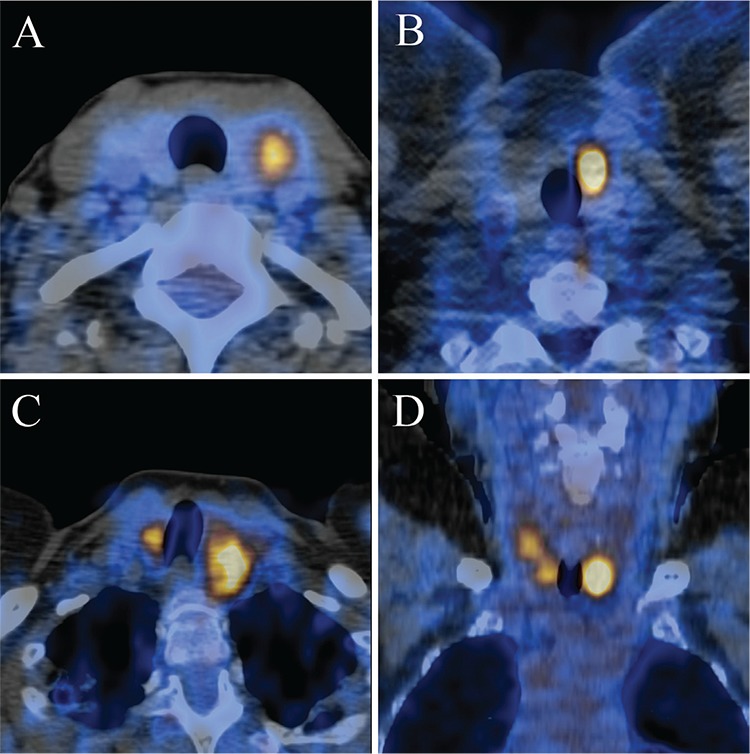
Four cases of incidental focal ^18^F-FDG uptake in the thyroid. Transaxial PET/CT fusion image of a benign left thyroid incidentaloma in a 52-year-old woman with a prior history of cervical cancer, with SUV_max_ 4.4, biopsied to reveal a benign follicular lesion (A), transaxial PET/CT fusion image of a malignant left thyroid incidentaloma in a 40-year-old man with a prior history of melanoma, with SUV_max_ 11.8, biopsied to reveal a papillary carcinoma follicular variant (B). Transaxial PET/CT fusion image of a bilateral focal benign thyroid incidentaloma in a 65-year-old woman with a prior history of lymphoma, with SUV_max_ 7.8 of left lesion and SUV_max_ 6.4 of right lesion, biopsied to reveal benign nodular hyperplasia (C), coronal PET/CT fusion image of a bilateral malignant thyroid incidentaloma in a 70-year-old woman with a prior history of colorectal carcinoma, with SUV_max_ 8.1 in the left lesion and SUV_max_ 4.5 in the right lesion, biopsied to reveal a multifocal papillary carcinoma, classical variant on a background of Hashimoto’s thyroiditis (D)

A receiver-operating-characteristic (ROC) curve analysis of sensitivities and specificities was performed to determine a clinically useful SUV_max_ cut-off value to aid in differentiating between benign and malignant lesions (Figure 1). PET/CT image examples of four patients with incidental thyroid uptake and their biopsy results, are provided in Figure 2.

## DISCUSSION

Incidental and unexpected focal uptake of ^18^F-FDG in oncologic PET/CT studies has been well studied in anatomic locations such as the breast, adrenal, gastrointestinal, parotid, prostate and thyroid glands (1,2,3,4,5,6,7,8,9,10). The thyroid gland is probably the best studied location of incidental ^18^F-FDG uptake on PET/CT, however, it remains a source of significant controversy. In recent years, three systematic reviews have been published, examining thyroid incidentaloma literature over the past 15 years, including an analysis of 18 papers by Shie et al. (11), 22 papers by Soelberg et al. (12), and 27 papers by Bertagna et al. (13). Although these three meta-analyses showed similar conclusions, the issue of the clinical significance of ^18^F-FDG positive thyroid incidentalomas has not been settled.

In our review of 7252 consecutive oncologic PET/CT studies, incidental focal thyroid ^18^F-FDG uptake was identified in 2.2% (n=157) of patients. This is within the range reported in the literature, with reports ranging from 0.2 to 8.9%. Shie et al. (11) reported 1% in 55,160 PET studies, Soelberg et al. (12) reported 1.6% in 125,754 PET studies and Bertagna et al. (13) reported a pooled incidence of 2.5% in 147,505 PET studies.

The rate of malignancy of thyroid incidentalomas varies tremendously in the literature from 10% to 64% (14,15) and remains a source of controversy. The rate of malignancy for our cohort was 10.9%. The mean malignancy rate was reported as 33.2% in the Shie et al. (11) review, as 34.8% by Soelberg et al. (12), and as 34.6% by Bertagna et al. (13). The concordance of these meta-analysis results is not surprising as many of the same thyroid incidentaloma papers were examined by all three reviews. However, these meta-analyses did not examine possible reasons why thyroid incidentaloma malignancy rates varied so much in the literature (10 to 64%).

Most published studies included in the three meta-analyses did not have histopathologic correlation or clinical follow-up on the majority of thyroid incidentaloma patients. In fact, the reported malignancy rates were usually calculated from a subset of biopsied patients who were biopsied most likely due to a high clinical suspicion of malignancy, which yielded, unsurprisingly, high malignancy rates. Soelberg et al. (12) reported a pooled biopsy rate of 46% (923/1994), and Shie et al. (11) reported a biopsy + follow-up rate of 56% (322/571). The largest meta-analysis by Bertagna et al. (13) reported a biopsy rate of only 35% (1308/3727) and noted that in the majority of the studies, the proportion of ^18^F-FDG positive thyroid incidentalomas that had further investigations was “inferior”. Shie et al. (11) expressed that the malignancy rate in the 44% of thyroid incidentalomas that were not investigated would be similar to the malignancy rate of those who were biopsied because of similar demographic characteristics in the two groups. This assumption is flawed. If the patients chosen for biopsy had been chosen randomly, then the assumption may have had merit, but biopsied patients were not chosen randomly. Studies generally do not provide any explanations of how or why certain patients were chosen for biopsy, but it is reasonable to assume that those selected for biopsy had a high clinical or imaging suspicion for malignancy. Soelberg et al. (12) admitted in their meta-analysis: “One cannot exclude that surgical confirmation was most likely obtained in those patients with the highest likelihood of malignancy and therefore the malignancy risk of focal uptake is overestimated”. We suspect that the reported average malignancy rate of 35% in the literature is overestimated and that the actual value is significantly lower.

An overview of the largest meta-analysis done by Bertagna et al. (13) (27 studies) reveals that the lowest malignancy rates are reported by studies with the highest biopsy rates. This pattern has not been noted either by Bertagna et al. (13) or the other two meta-analyses. We examined all papers with a biopsy rate of over 80% and further analyzed the available data (Table 7). Studies by Chen et al. (16) and Ohba et al. (21) were excluded as they were done on healthy volunteers only, and the study by Zhai et al. (19) was excluded as the patient population was unspecified. This leaves only four studies of oncologic patients with thyroid incidentalomas with high biopsy rates of more than 80%. These studies showed malignancy rates of 10% (14), 14% (17), 23% (20) and 24% (22). It is worth noting that the only North American study published in the literature with a high biopsy rate showed a malignancy rate of 14% (17), very close to our rate of 11%.

In our cohort of 128 thyroid incidentaloma patients, there was a statistically significant difference between the SUV_max_ values of benign thyroid incidentalomas (median SUV_max_ 4.8) and malignant incidentalomas (median SUV_max_ 6.3), however, there was a wide overlap of SUV_max_ values between the two groups. An ROC curve was generated, however no suitable SUV_max_ cut-off value was found to be useful in differentiating benign from malignant thyroid incidentalomas (Figure 1). This is in agreement with all three meta-analyses, all of which found a statistically significant difference between SUV_max_ of benign lesions vs malignant lesions, with a wide overlap and no clear SUV_max_ cut-off value, or role for the use of SUV_max_ to differentiate benign from malignant thyroid incidentalomas.

In our clinically followed group (71 of 128 patients), 29 of 71 (41%) patients also had a follow-up PET/CT and incidental thyroid uptake was re-evaluated on the follow-up PET/CT. Interestingly, although all 29 patients were determined to have benign thyroid incidentalomas on long term clinical follow-up, 8 of 29 (28%) follow-up PET/CTs showed increased SUV_max_ in the thyroid incidentaloma (defined as any increase over the previous SUV_max_ value), with the rest showing equal or lower SUV_max_, suggesting that increasing SUV_max_ on a follow-up PET/CT may not be helpful in assessing whether a thyroid incidentaloma was benign or malignant, and therefore a follow-up PET/CT is unlikely to be a useful imaging modality to monitor and follow thyroid incidentaloma patients. Further research in this area is needed to determine the optimal management of thyroid incidentaloma patients.

### Study Limitations

A limitation of our study was that only 57 of 128 thyroid incidentaloma patients (45%) were biopsied. Ideally, a thyroid incidentaloma study would be prospective and all ^18^F-FDG positive focal thyroid incidentalomas would have biopsy results available. However, unlike most studies that had not evaluated or followed thyroid incidentaloma patients who were not biopsied, our 71 patients who were not biopsied were followed for at least 12 months.

## CONCLUSION

^18^F-FDG positive focal thyroid incidentalomas occurred in 2.2% of oncologic PET/CT scans, and of these, 10.9% were malignant. This is the lowest malignancy rate reported in a North American study and second lowest in the world to date, and is much lower than the average 33-35% malignancy rate reported in recent systematic reviews. Higher reported malignancy rates in the literature may be the result of selection bias. The decision to biopsy a thyroid incidentaloma should be deferred in the absence of a high clinical or imaging suspicion of malignancy. Recommendations to biopsy all ^18^F-FDG positive focal thyroid incidentalomas should not be followed until further research is available. We suspect that the true malignancy rates of thyroid incidentalomas are in the 10-20% range, rather than 35% (or higher) range which is often quoted in the literature. SUV_max_ values cannot be used to differentiate benign from malignant thyroid incidentalomas, and follow-up PET/CT may not be useful in monitoring these patients. Future studies should be prospective and biopsy rates should be as high as possible, to avoid selection bias that may significantly impact reported malignancy rates.

## Figures and Tables

**Table 1:**
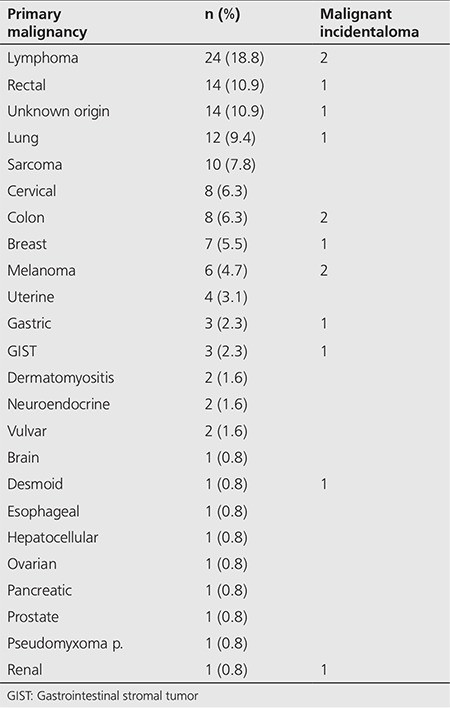
Study population (n=128)

**Table 2:**
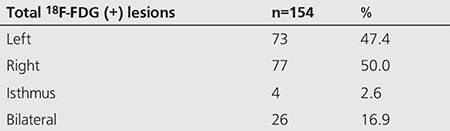
Location of thyroid incidentalomas

**Table 3:**
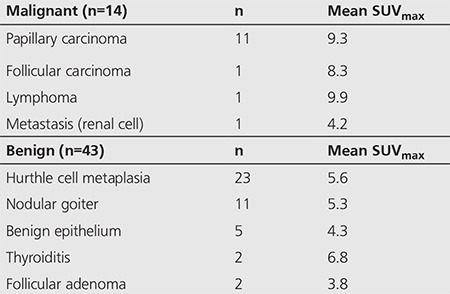
Biopsied thyroid incidentalomas (n=57)

**Table 4:**
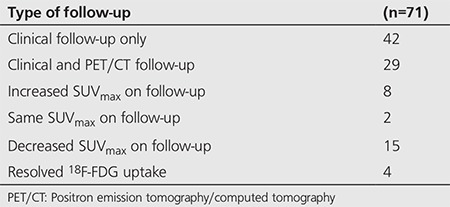
Clinical follow-up

**Table 5:**
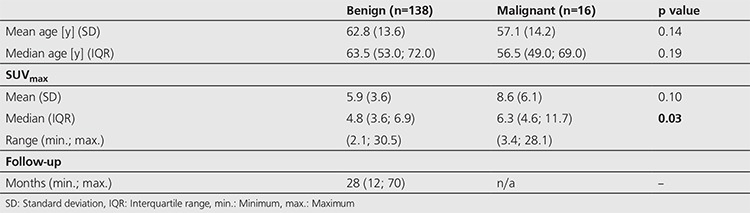
Thyroid lesion analysis

**Table 6:**
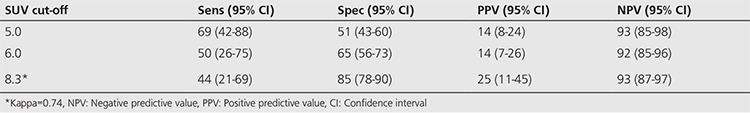
Malignancy vs. SUV_max_ cut-off (Receiver-operating-characteristic)

**Table 7:**
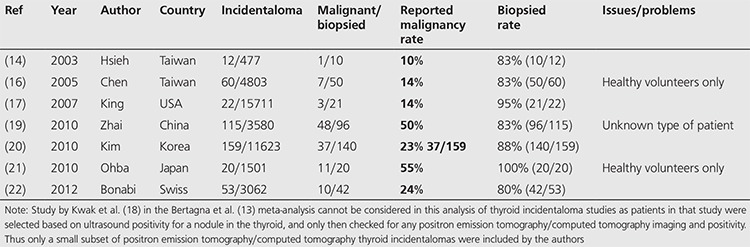
Thyroid incidentaloma studies with biopsy rates >80%
